# A Systematic Review of Fibonacci Sequence in the Human Abdominal Wall: Facts and Reality

**DOI:** 10.7759/cureus.33072

**Published:** 2022-12-28

**Authors:** Chetna Ravindra, Vinayak Rengan, Ramana B

**Affiliations:** 1 General Surgery, Madras Medical College, Chennai, IND; 2 General Surgery, Dr. Mehta's Hospital, Chennai, IND; 3 Institute of Minimal Access, Metabolic and Bariatric Surgery (IMAS), Sir Ganga Ram Hospital, New Delhi, IND; 4 Abdominal Wall Reconstruction, Clinic Ramana, Kolkata, IND

**Keywords:** hernia, descriptive anatomy, functional anatomy, umbilicus, abdominal wall reconsruction

## Abstract

The Fibonacci sequence is undoubtedly found in nature such as in the spiral of galaxies and flower petals. Fibonacci numbers are a sequence in which each number is the sum of the two preceding ones. The ratio of two consecutive Fibonacci numbers, also called the golden proportion, approximately equals 1.618. We analyzed the existence of Fibonacci numbers and golden ratios in the field of hernia and abdominal wall reconstruction. We found substantial evidence of the use of the golden ratio in siting of the umbilicus. The Fibonacci numbers also showed up frequently in the anatomy of the abdominal wall. However, this was not as appropriate as the other instances in the human body or in nature.

## Introduction and background

The mystical arrangement of rose petals, shells, sunflower seeds, and even the galaxies in outer space has been a source of fascination for mathematicians and scientists alike because they tend to follow the order of a specific number pattern or proportion originally described by the 13th-century Italian trader-mathematician known as Fibonacci [1,2]. These numbers form a sequence known as the Fibonacci sequence such that each number is the sum of the two preceding ones. The Fibonacci sequence goes like this: zero, one, one, two, three, five, eight, 13, 21, 34, 55 [1]. The ratio of any two sequential Fibonacci numbers approximates the value of 1.618, commonly represented by the Greek letter phi, φ. The value is closer to the actual value of phi when the numbers are higher and is unclear with lower value Fibonacci numbers.

Many famous architects incorporated the divine proportion in their work. The Parthenon and the Great Pyramids of Giza are famous examples [3]. Marcus Vitruvius was a connoisseur of perfect ratios in nature and drew parallels between buildings and the human body, measuring every imaginable distance in the body and calculating ideal proportions [4]. Leonardo da Vinci furthered his work [4]. In his famous image of the proportions of the human body, he shows that we fit into both circles and spheres, following perfect geometric forms.

Fibonacci numbers and the golden ratio have been found in multiple ratios of the human body, as described by Ernst Neufert [5]. It has been remarkable to find further applications in the human body at the cellular and molecular levels, such as the branching of lung bud during embryogenesis, arrangement of nucleic acid bases in the spiral of DNA, and even in colon stem cell turnover [6-8]. We aimed here to analyze and discover if this pattern exists in the field of hernia and abdominal wall reconstruction surgery. Most numbers and sequences in the anterior abdominal wall appear in sequences of two, three, or five which are Fibonacci numbers.

## Review

Methods

We performed a literature search using the keywords "Fibonacci sequence," "Fibonacci numbers," "golden ratio," "abdominal wall," "hernia," "abdominal anatomy," and "abdominal wall reconstruction" used in various combinations on PubMed, PubMed Central, and Google Scholar up to October 20, 2022. The search results, shown below in Table 1, were then evaluated to extract information about the occurrence of Fibonacci numbers in the anatomy of abdominal wall and its reconstruction for abdominal wall hernia. Articles that were not related to Fibonacci sequence in the field of abdominal wall reconstruction were excluded. All references of included articles were also analyzed.

**Table 1:** Search strategy and results.

Search Strategy	Database	Results
(Fibonacci sequence OR Fibonacci numbers OR golden ratio) AND (abdominal wall OR hernia OR abdominal anatomy OR abdominal wall reconstruction)	PubMed	16
(Fibonacci sequence OR Fibonacci numbers OR golden ratio) AND (abdominal wall OR hernia OR abdominal anatomy OR abdominal wall reconstruction)	PubMed Central	2996
"Fibonacci sequence" "Fibonacci numbers" "golden ratio" "abdominal wall" "hernia" "abdominal anatomy" "abdominal wall reconstruction” - used in combinations	Google Scholar	409
-	Total	3421

Inclusion and Exclusion Criteria

All articles conducted on humans older than 18 years in English language, or with official English translations were included. Studies not in English language and animal studies were excluded from the study.

Outcomes of Interest

The occurrence of Fibonacci numbers in the anatomy of the abdominal wall and its reconstruction for abdominal wall hernia was deemed as the primary outcome. All articles and their references were also scoured for the novel identification of a pattern of Fibonacci numbers.

Quality Assessment

The following means were used for quality appraisal of the studies: Newcastle-Ottawa scale for observational studies and Scale for the Assessment of Narrative Review Articles (SANRA) checklist for narrative reviews and book chapters [9,10]. A benchmark of seven stars on the Newcastle-Ottawa scale and 70% on SANRA checklist was established to qualify for review.

Results

**Figure 1:**
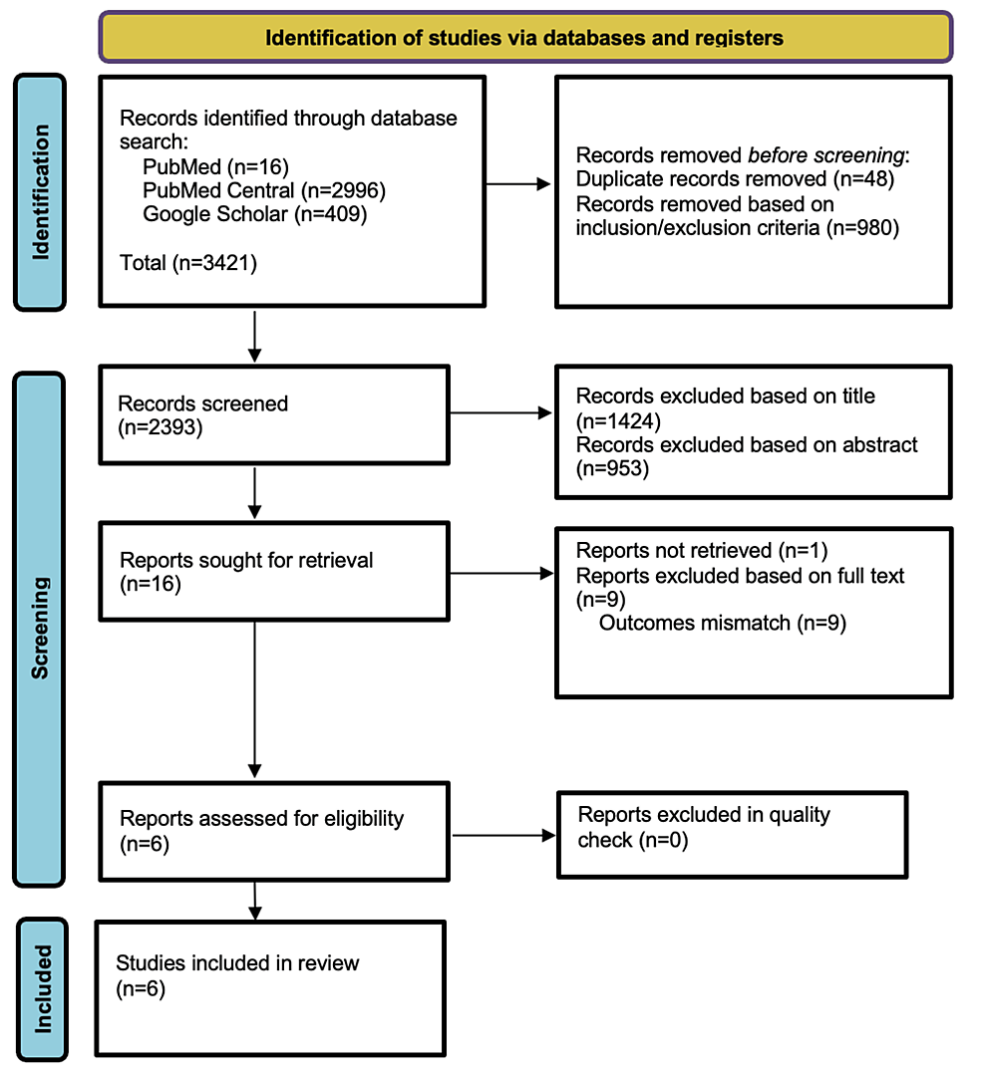
Preferred Reporting Items for Systematic Reviews and Meta-Analyses (PRISMA) flowchart. PRISMA 2020 flowchart showing study selection [11].

A total of 3421 studies were identified through database search as shown in Figure 1. After the exclusion of 48 duplicates, articles were selected by inclusion and exclusion criteria. A total of 2393 articles were then screened by title and abstract. 16 articles were retrieved for full text. One could not be retrieved. Articles with different outcomes were excluded. Six studies then underwent quality appraisal to finalize the articles for review.

Study Selection

Our systematic review included six articles. The included studies and their characteristics are shown below in Table 2 [12-17].

**Table 2:** Studies selected and their characteristics. SANRA: Scale for the Assessment of Narrative Review Articles

Number	Study	Year	Type of article	Quality assessment
1	Bueno-Lledó et al. [12]	2018	Observational study	Newcastle-Ottawa scale
2	Majumder [13]	2016	Book chapter	SANRA checklist
3	Visconti et al. [14]	2016	Observational study	Newcastle-Ottawa scale
4	Visconti et al. [15]	2014	Observational study	Newcastle-Ottawa scale
5	Avisse et al. [16]	2000	Narrative review	SANRA checklist
6	Davis et al. [17]	1979	Observational study	Newcastle-Ottawa scale

Discussion

In the study of the human body, golden ratios have historically been the focus of many physician-scientists with new ratio patterns being discovered in the lengths of phalanges [18], facial esthetic ratios [19], and even cardiac cycle systole-diastole ratios [20]. The golden proportion is also touted for the perfect smile, with the width ratios of the upper jaw teeth being 1.618, 1, and 0.618 [21].

The Umbilicus

Davis et al. in 1979 measured various distances from the umbilicus for 252 Indian and 207 German students [17]. They found the ratio of the distance from umbilicus to foot to the distance from the umbilicus to head equaled the golden ratio [17]. The number showed up again in the ratio between total height to the distance from umbilicus to foot. This is the only observational study done to measure the location of the umbilicus with respect to entire length of the body and had significant results.

**Figure 2:**
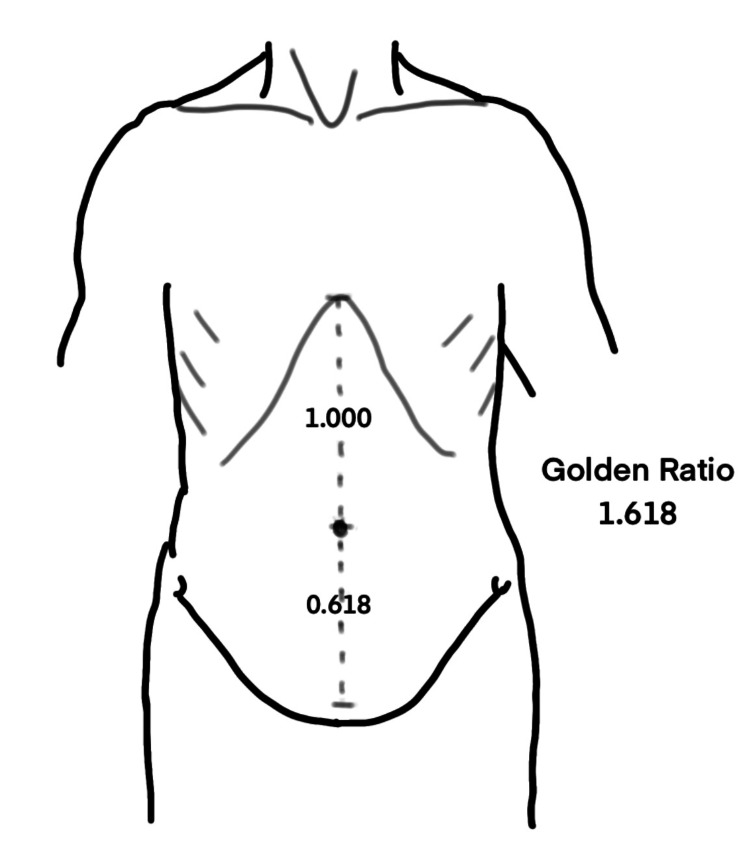
Golden ratio in the positioning of the umbilicus. The illustration is created by the author (C. Ravindra) of this study.

**Figure 3:**
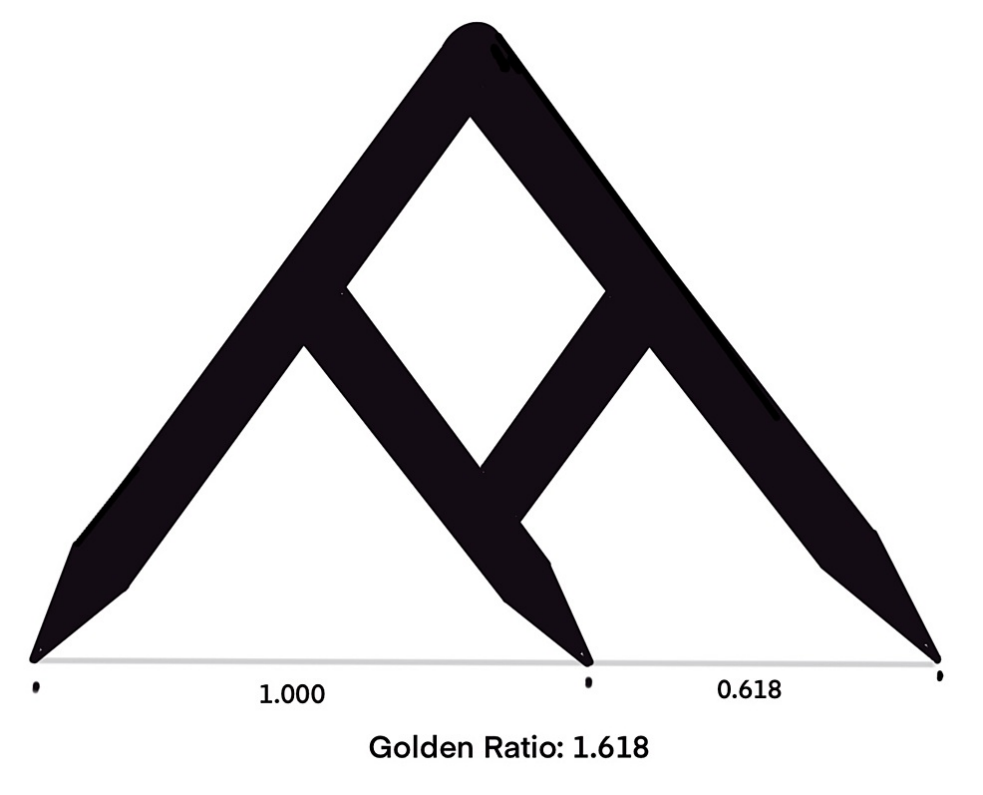
Representation of the Fibonacci calipers. The illustration is created by the author (C. Ravindra) of this study.

The umbilicus seems to have a golden role, beyond its central role in fetal life. Visconti et al. measured the xipho-umbilical distance and the umbilicus-to-abdominal crease distance or umbilicus crease distance in 81 models [15]. This study showed that a golden ratio existed in the ratio between the xipho-umbilical distance and the umbilicus-to-abdominal crease distance. An analysis of 1682 online volunteers who rated the images of models revealed that the umbilicus is considered attractive when located in the golden ratio, i.e., the xiphoid-umbilicus to umbilicus-abdominal crease ratio was 1.618. This principle has potentially important applications in siting or positioning the umbilicus after abdominal wall or cosmetic surgery further described by Visconti et al. in another article [14]. A Fibonacci caliper might aid in the proper positioning of the umbilicus on the abdominal wall and help in avoiding an undesirably high- or low-riding umbilicus [14]. The golden ratios in umbilical positioning and Fibonacci calipers are shown in Figures 2, 3.

The Abdominal Wall Layers

**Figure 4:**
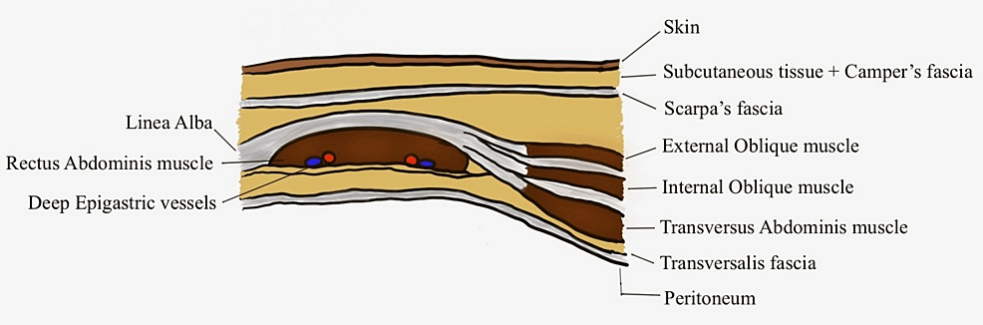
Section of the abdominal wall below the arcuate line. The illustration is created by the author (C. Ravindra) of this study.

The superficial fascia of the anterior abdominal wall has two layers - Camper’s and Scarpa’s fascia. There are two layers of muscle-derived fibers that cover the rectus muscle anterior and posterior, cranial to the arcuate line. There are three layers that cover the rectus muscle anteriorly, caudal to the arcuate line [13]. Figure 4 shows the section of the abdominal wall below the arcuate line.

There is one central linea alba that divides the two rectus muscles. Each of the rectus muscles has three tendinous intersections on the rectus abdominis [22]. There are three lateral muscles on the anterior abdominal wall - the external oblique, internal oblique, and the transversus abdominis on either side. There are also two recti on either side. The total number of muscles is eight (2 + 3 + 3). Hence, the layers in the abdominal wall present in groups of two or three, are also Fibonacci numbers.

Peritoneal Spaces

**Figure 5:**
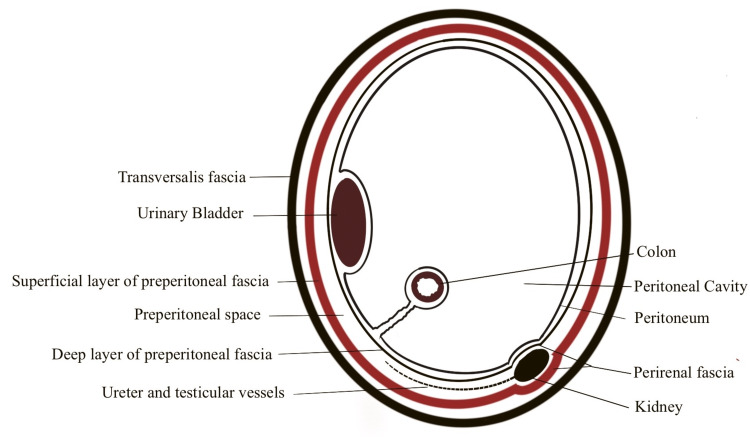
Preperitoneal space and preperitoneal fascia. The illustration is created by the author (C. Ravindra) of this study.

There are two named extra-peritoneal spaces - the lateral space of Bogros and the medial space of Retzius. The apparently lone preperitoneal space is divided by two preperitoneal fasciae into three subspaces - superficial space, true-preperitoneal space, and deep space as described by Sakurai [23]. Figure 5 shows the preperitoneal fascia and the division of the preperitoneal space.

Neurovascular Supply

There are three vascular zones in the anterior abdominal wall [13]. "Zone 1" or the medial zone covers the upper anterior abdominal wall. "Zone 2" or the caudal zone covers the caudal portion of the anterior abdominal wall. "Zone 3" or the lateral zone is situated laterally beyond the linea semilunaris [13].

There are five cutaneous abdominal nerves in total. The anterior divisions of the seventh, eighth, ninth, 10th, and 11th thoracic intercostal nerves supply the rectus abdominis muscle and terminate as anterior cutaneous branches of the abdomen supplying the cutaneous sensation to the anterior abdominal wall. The inguinal area further has three nerves of interest - the iliohypogastric nerve, the ilioinguinal nerve, and the genital branch of the genitofemoral nerve from cranial to caudal [24].

Laparoscopic View

While viewing the abdomen through a laparoscope, there is one obliterated urachus in the center, two ligaments on the left (left medial and lateral umbilical ligaments), and two ligaments on the right (right medial and lateral umbilical ligaments). So, overall, there are five ligaments. Each side has three spaces - supravesical fossa, medial inguinal fossa, and lateral inguinal fossa [16].

The triangle of doom has - two nerves (the genital branch of the genitofemoral nerve and, hidden by fascia, the femoral nerve); three borders (the vas deferens medially, spermatic vessels laterally, and peritoneal fold inferiorly); and three vessels (external iliac artery, external iliac vein, and deep circumflex iliac vein) [16].

**Figure 6:**
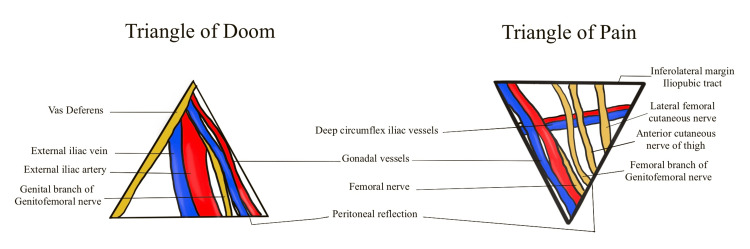
Triangle of doom and triangle of pain. The illustration is created by the author (C. Ravindra) of this study.

The triangle of pain contains three nerves - the lateral femoral cutaneous nerve, femoral nerve, and the genitofemoral nerve [16]. The triangles of doom and pain are demonstrated in Figure 6.

Classification of Hernia

The classification of hernia by the European Hernia Society (EHS) also shows Fibonacci numbers. The EHS classification for midline incisional hernias has three components, which are classified into five levels (M1-M5) each 3 cm apart (Table 3) [25]. The EHS classification for parastomal hernias has two categories, small <5 cm and large >5 cm [26].

**Table 3:** European Hernia Society classification for midline incisional hernias.

EHS incisional hernia classification		
Midline	Subxiphoidal	M1
	Epigastric	M2
	Umbilical	M3
	Infraumbilical	M4
	Suprapubic	M5
Lateral	Subcostal	L1
	Flank	L2
	Iliac	L3
	Lumbar	L4
Recurrent incisional hernia? Yes/No		
Length (cm)	Width cm	
W1	W2	W3
<4 cm	≥4-10 cm	≥10 cm

Botox for Preoperative Progressive Pneumoperitoneum

The use of Botox for preoperative progressive pneumoperitoneum described by Bueno-Lledó et al. also shows the Fibonacci numbers beautifully [12]. It involves one injection, the botulinum toxin A injected on two sides right and left. The following three muscles are injected: the external oblique, internal oblique, and transversus abdominis. Five points were identified on either side of the abdominal wall - two points in the mid-axillary line between the rib margin and the upper iliac crest, and three points between the anterior axillary line and midclavicular line between the costal margin and the superior iliac crest. The maximal effect is seen in two weeks and the effect lasts for eight months.

Occurrence in Plastic Surgery

Fibonacci sequence has been discussed abundantly in plastic and reconstructive surgery. An article by Bozola discusses 502 patients in whom abdominoplasty was done maintaining a golden ratio in the hip to waist circumference as well as umbilical positioning [27]. Outside of the abdomen, Fibonacci sequence has also been used in mammaplasty and breast reduction [28]. Perhaps the most widely used application has been in cosmetic surgery of the face. Ricketts described maintaining a golden ratio for width of upper lip to the width of the nose and also in temporal width of the face to the width of eyes measured between the two lateral canthi [29].

## Conclusions

The Fibonacci numbers are found aplenty in the abdominal wall anatomy and some surgical procedures. The most useful association is definitely in the location of the umbilicus in the abdomen. The clinical application includes positioning of the umbilicus postabdominal wall reconstruction by maintaining a ratio of 1.618 between the xipho-umbilical distance and the umbilicus-to-abdominal crease distance. However, we also agree that the correlation is not as fulfilling as the mystical pattern in facial and smile esthetics, systole-diastole ratio, or lung bud branching during embryogenesis. Further studies may be ideal to discover further instances of the Fibonacci sequence in the abdominal wall. Implementation of umbilicus positioning in the golden ratio needs further examination by clinical trials.
